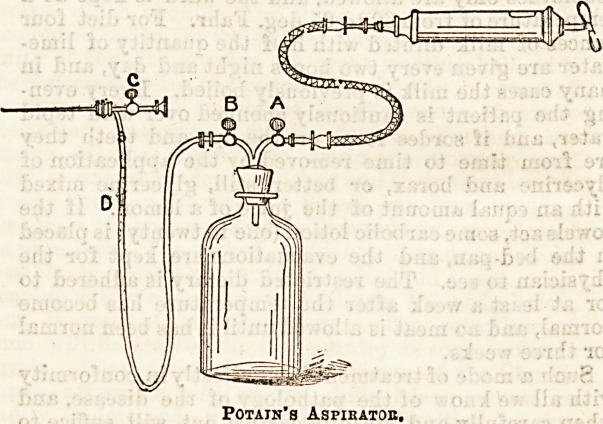# The Treatment of Pleurisy

**Published:** 1892-12-10

**Authors:** 


### ROYAL FREE HOSPITAL.

### The Treatment of Pleurisy.

In the treatment of simple acute pleurisy at the Royal Free Hospital, the patient is, of course, confined to bed, and the diet is restricted to liquids, consisting generally of milk and beef-tea. The bowels are opened either by pil. colocyn. c̄. hyoscy., or by a draught of the hospital mixture, haustus sennæ co. A simple saline such as the following is often prescribed to act upon the skin: ℞. liq. ammon. acet. ʒij.; acidi nitrohydroch. dil ʒiss.; aq. ad. ℥viij.; ℥j. 4tis. horis ex. aq. And if the pain be severe, opium, generally in the form of the tincture, is administered.

Supposing that the amount of fluid poured out into the pleural cavity be not excessive, and that absorption go on steadily and satisfactorily as it does in most cases, no active treatment is adopted, though occasionally when the acute stage is over the patient is given some iodide of potassium to hasten the process. But if there are signs that the presence of the fluid is embarrassing the heart's action, or if, apart from this, absorption is not progressing and there is danger of permanent damage to the lung from the continued pressure, then the question of paracentesis thoracis comes up, and unless there be any strong contra-indication, it is performed. As regards the site for the puncture, it of course varies with the position of the fluid. If the pleurisy be encysted the needle is entered about the middle of the collection; but in ordinary cases in which the fluid is free in the cavity, Dr. West prefers a point in the mid-axillary line in the sixth space, just above the seventh rib. The selected site is carefully percussed and auscultated to make sure that there is no adherent lung, and that in the case of left-sided pleurisy the heart runs no danger of being injured, though of course that is usually pushed over by the fluid to the other side. As a preliminary, Dr. West usually removes a little fluid with a small aspirator, but always with strict antiseptic precautions, the needle being first boiled for some minutes to ensure perfect cleanliness; and if the result of this exploratory puncture is satisfactory, the bulk of the fluid is drawn off. The patient is placed in a recumbent position near the edge of the bed with the shoulders slightly raised, and rather inclining to the diseased side, and the skin over the site of the proposed puncture is well cleansed with a solution of carbolic acid.

The following is a description of the apparatus used for this operation at the Royal Free: There is an indiarubber cork which fits any ordinary bottle; this is pierced by two tubes*, the shorter (a) being connected with an exhausting syringe, and the other (b) with the cannula. This one is interrupted near the cannula by a piece of glass tubing (d) let in, so that the nature of the fluid, the rate of flow, &c., may be observed; both tubes are provided with stopcocks. The trocar and cannula are boiled to render them aseptic, the bottle is placed on the floor, stopcock (a) being turned off; the skin over the place chosen for puncture is stretched between the thumb and finger, and the needle introduced with a quick movement. The trocar is withdrawn, and the fluid flows along the cannula and tube into the bottle. When the flow lessens because of diminution of pressure in the pleural cavity, stopcock (b) is turned off, and (a) turned on, and the air in the bottle rarefied by a few strokes of the exhausting syringe; a and b are now reversed, when the fluid generally flows freely again for a time. If necessary this is repeated a few times, care being taken not to use too great suction power. Sometimes the mouth of the cannula becomes blocked with fibrin or *débris*; in this case the cannula is shifted about a little, or the stilette is again introduced to clear the passage. The fluid is drawn off slowly till no more flows, or till it is seen to be blood-stained, and then the

Potain's Aspirator.

cannula is withdrawn, the edges of the wound being compressed between the thumb and finger, and the opening closed by the application of small pieces of wool soaked in collodion; the side is kept at rest by a broad piece of strapping firmly applied and reaching beyond the middle line both in front and behind. If the patient becomes at all faint during the flow he is given a little brandy. After the withdrawal of part of a pleuritic effusion the rest is often quickly absorbed, even when a good deal is left in the pleural cavity. Occasionally the operation has to be repeated once or twice.

In cases where the fluid effused is purulent, or has become so after paracentesis, the treatment varies. Sometimes the pus is removed by an aspirator, and the opening closed, but more often an incision is made, and when as much pus as will do so has escaped, a piece of drainage tube three to five inches long is introduced, through which, probably, more fluid will at once escape. A loop of silk or thread is attached to the tube, and carefully fastened to the skin with a piece of strapping, to avoid the repetition of a disaster of which the traditions of the hospital speak. The side is washed and the wound generally dusted with iodoform. The dressing consists of, first, several layers of sal alembroth gauze dipped in perchloride of mercury, then dry gauze, the whole being covered with plenty of sal alembroth wool, and a bandage applied.

Sometimes, especially in children, a piece of rib has to be resected, as otherwise a sufficiently large opening for the free evacuation of the pus cannot be made.

There have been a few cases at the Royal Free of chronic empyema, where the patient was emaciated and exhausted by the constant discharge, and the lung has been so compressed and bound down that a large cavity existed between the chest-wall and the lung; and in one or two instances Estlander's operation has been performed in the hope that the chest-wall would then fall in, and the size of the suppurating cavity be diminished, if not obliterated. One patient had pieces

* For practical convenience these two tubes may be combined while in fact remaining two distinct tubes, as shown in the figure.

removed from four or five ribs, but the result was not very satisfactory, the condition of the patient being such that the wounds would not heal.

In the out-patient department many cases of dry pleurisy are met with, where there are no constitutional signs, and the pain complained of is due to the friction of the inflamed and tender surfaces of the pleura. Here the painful part is generally painted with iodine, and the affected side strapped to limit the movement.

## Figures and Tables

**Figure f1:**